# Insights and limitations of endometrial cancer risk prediction models for clinical applicability: a systematic review

**DOI:** 10.1186/s12885-025-15200-x

**Published:** 2025-11-19

**Authors:** Sabine El-Halabi, Alison Zhijin Luo, Aline Talhouk

**Affiliations:** Department of Obstetrics and Gynecology, Faculty of Medicine, University of British Columbia, 5th Floor (593), VGH Research Pavilion 828 West 10th ave, Vancouver, BC V5Z 1M9 Canada

**Keywords:** Endometrial cancer, Prediction, Risk models, Prevention, Risk factors, Incidence, Discrimination, Calibration

## Abstract

**Background:**

Endometrial cancer (EC) is the most common gynecologic cancer in high-income countries, with rising incidence rates. Risk prediction models can identify high-risk individuals, enabling targeted prevention and early intervention. Despite the development of several multivariable risk models aimed at stratifying EC risk, none have yet been adopted for clinical use in cancer prevention. This systematic review critically examines the performance, validation, and clinical applicability of existing EC risk prediction models.

**Methods:**

We systematically searched online search engines PubMed and Ovid MEDLINE for EC risk model publications written in English from January 1, 2000, to October 9, 2024. Studies were selected based on the inclusion of multivariable models for EC risk estimation. Data extraction focused on cohort characteristics, predictors included, validation efforts, and model performance metrics such as discrimination (C-statistics or AUROC) and calibration (E/O ratio or calibration slopes). The quality of model reporting was assessed using the TRIPOD-AI guidelines.

**Results:**

Nine risk prediction models were identified, predominantly based on epidemiological factors, with four incorporating polygenic risk scores, and one using blood biomarkers. Most models were developed in datasets of postmenopausal women of White or European ancestry from Western countries. Only five models were externally validated; most exhibited moderate discrimination (AUROC ranging from 0.64 to 0.77). Calibration varied, with some models showing significant overestimation of risk. Importantly, the lack of racial and ethnic diversity in the development datasets limits their generalizability, particularly for non-White populations.

**Conclusions:**

Current EC risk prediction models show moderate performance but suffer from limited external validation, homogeneity in demographics, and exclusion of diverse populations. Future research should focus on broadening participant diversity and incorporating new risk factors, such as hormonal intrauterine device use, hysterectomies, environmental exposures, and socio-economic status. Developing dynamic models that account for these factors and model outcomes that span various forms of the disease can improve clinical relevance. Personalized, risk-based approaches targeting high-risk groups may offer a viable path forward for EC screening and prevention strategies, ensuring more equitable cancer care and improving patient outcomes.

**Supplementary Information:**

The online version contains supplementary material available at 10.1186/s12885-025-15200-x.

## Introduction

The incidence of endometrial cancer (EC) continues to rise globally, particularly in high-income countries, where it is the most common gynecological malignancy and the fourth most common cancer among females [1, 2]. In North America, EC rates have increased annually by approximately 1% among White women and 3–6% among women from other racial/ethnic groups [3–6]. This trend is projected to continue [7], largely driven by rising obesity levels [8, 9], a well-established risk factor for EC [10]. Obesity rates are higher in individuals with lower socio-economic status (SES) and may inequitably impact black and ethnic populations [11]. Mortality rates have followed a similar upward trajectory, increasing by 1.6–1.9% annually over recent years, disproportionately impacting Black women and individuals from under-represented ethnicities [5, 12, 13]. The increase in mortality has been attributed, at least in part, to diagnoses of more aggressive forms of the disease [13–15].

If detected while confined to the uterus, the five-year survival rate of EC exceeds 95% [16]. However, if the cancer spreads beyond the uterus, this rate drops to 18%, underscoring the importance of early detection [16]. There are no universal screening recommendations for EC [17, 18], except for people with Lynch syndrome, a genetic condition that increases the risk of EC to 20–60% [19] compared to a 3% risk in the general population [20]; regular transvaginal ultrasounds, endometrial biopsies, and hysterectomies have been endorsed for this group [21, 22]. Besides genetic predisposition, about 40% of EC cases can be attributed to modifiable risk factors [17, 23], including obesity [24], reproductive factors [25], and hormonal exposures [26]. Prevention strategies such as physical activity, weight loss, bariatric surgery, and hormone interventions have been demonstrated to be effective at reducing EC risk [23–28]. Accurately identifying high-risk individuals can help target these interventions to those most likely to benefit, reducing associated harm and making them more cost-effective.

Given the complex interplay between risk factors associated with EC, relying on individual factors alone to assess risk may not be adequate. To address this, multivariable predictive models [29–37] have been developed to estimate the individual absolute risk of developing EC over a specific period, incorporating a range of epidemiological data, genetic factors, and biological factors [38]. The choice of the dataset used for model development influences which risk factors are included in the model, and the composition of the cohort can potentially impact the model’s generalizability across diverse populations. Additionally, a lack of transparency in reporting can hinder future validations and broader clinical applications.

This review critically assessed previously developed EC risk models by examining the populations studied, the predictors used, and the methodologies employed. We compared models’ performance and analyzed their strengths, weaknesses, and limitations, offering a foundation for future model validation and improvement of these models to impact prevention, early detection, and bridge inequities in EC.

## Methods

### Literature search

We closely adhered to the Preferred Reporting Items for Systematic Reviews and Meta-Analyses (PRISMA) guidelines (Additional File 1) to report the methods and results in this systematic analysis [39]. Ethical approval was not required, as this study reviewed published literature and did not involve primary data collection. A protocol has been registered in the Open Science Framework [40]. The literature was searched for studies that developed models of any methodology to predict EC incidence in asymptomatic populations. We searched PubMed and Ovid MEDLINE databases for English-language papers published between January 1, 2000, and October 9, 2024, using the following medical search heading (MeSH) terms: “endometrial neoplasms” and “risk factors” with various keywords including “asymptomatic”, “prediction”, and “model”. Searches were conducted across multiple fields, including titles and abstracts. Details of the search are in Additional File 2. We also conducted a manual search for studies by reviewing the references of the articles included to capture any studies not identified in the database search.

### Eligibility criteria

To be included in the review, studies had to develop or validate risk prediction models for EC incidence in women without any symptoms or suspicion of cancer; they also had to include risk factors of any type (e.g., epidemiological, genetic, etc.), and report model performance metrics such as discrimination or calibration. We excluded primarily diagnostic and prognostic models, models predicting recurrence or survival, models predicting general cancer risk, or models not providing performance metrics. We excluded abstracts, conference proceedings, letters to the editor, books, and studies inaccessible in full text.

### Data extraction and quality assessments

Two authors (S.E.H. and A.Z.L.) systematically screened all titles and abstracts for eligibility in each database and then conducted a full-text review independently. Any disagreement was resolved through discussion and by including a third author (A.T.). Data extraction for development and validation characteristics from eligible studies included year of model development, study design, region, population characteristics (age, menopause status, ethnicity, and any specific subgroup considerations), inclusion and exclusion criteria, years of data collection, sample size and cancer cases, risk factors, model characteristics, performance metrics (Area Under the Receiver Operating Curve (AUROC), C-Statistic, Expected/Observed ratio (E/O), calibration slopes). The quality of the included studies was assessed according to the TRIPOD (Transparent Reporting of a Multivariable Prediction Model for Individual Prognosis or Diagnosis) guidelines [41]. The TRIPOD checklist evaluates clarity in model development or validation, the rationale for and purpose of the model outlined in the introduction, detailed methods, population selection, and statistical analysis. It also checks whether key metrics such as calibration and discrimination are reported and if the model’s strengths, limitations, and potential bias are reported. Each section was rated as low, medium, or high quality. Models that met more than 75% of the criteria were considered high quality, and the overall rating was based on the majority of section scores. Two authors (S.E.H. and A.Z.L.) navigated these checklists and extracted key elements such as sample size, inclusion and exclusion criteria, modelling strategies, completeness of reporting, internal and/or external validation, and handling of missing data. Studies that did not adhere to reporting standards or were insufficiently validated were noted in the analysis. Any limitations or inconsistencies in performance were highlighted.

## Results

### Search results and study characteristics

The initial search yielded 2,633 studies from PubMed and 3,637 studies from MEDLINE (Ovid); After removing duplicates and screening titles and abstracts, 29 full-text articles were reviewed for eligibility. Of these, 21 studies were excluded: Six were diagnostic, six were prognostic, four focused on symptomatic individuals, three did not develop multivariate risk models, and two did not predict EC incidence. An additional study was identified through a manual reference search. The study selection flow diagram is provided in Additional Fig. 1.

The nine studies (Additional File 3, Table S1) that met our eligibility criteria developed models to predict EC incidence in a population without cancer or cancer symptoms [29–37]. Four studies focused exclusively on epidemiological risk factors [29–31, 34]. The earliest model, developed by Pfeiffer et al. [29] in the United States (US), was followed by a similar model by Hüsing et al. [30], using data from ten European countries. In 2017, Fortner et al. [32] enhanced the Hüsing [30] model by incorporating serum biomarkers as additional predictors. In 2020, Hart et al. [31] used the same dataset as Pfeiffer et al. to propose machine learning (ML) algorithms for predicting EC risk. In 2020, several models began considering genetic markers, including single nucleotide polymorphisms (SNPs) [33, 35–37]. In 2023, Kitson et al. [34] proposed a new epidemiological model using data from the United Kingdom (UK). In the same year, Shi et al. [33] developed two models using a dataset which spanned the US, Alberta (Canada), Australia, and Europe. The details of the models are presented in Additional File 3, Table S2.

### Datasets for model development

**Fig. 1:**
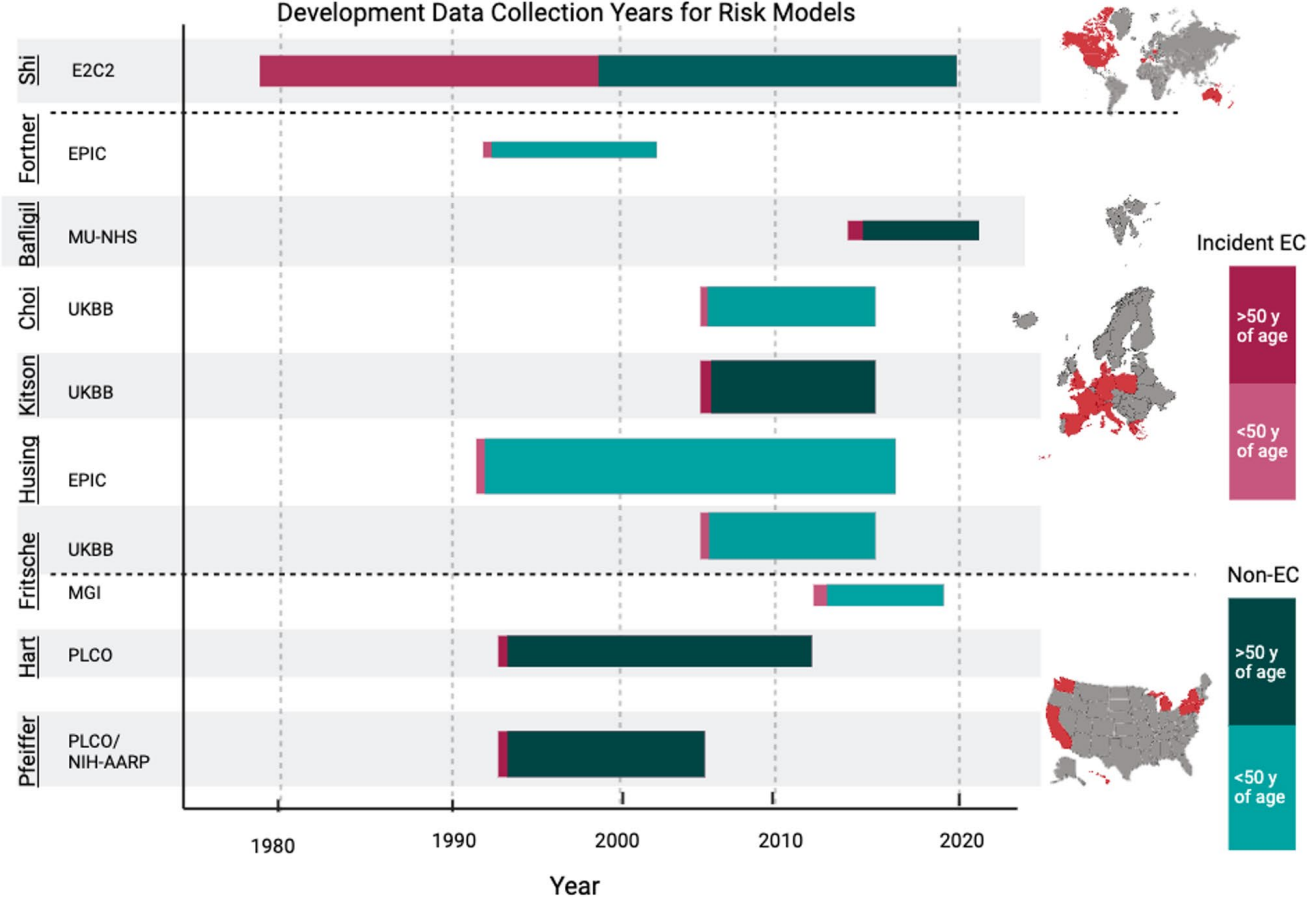
Development data collection years for each risk model by dataset. Graphical representation of the data collection span, from recruitment years to estimated follow-up, for each dataset used to develop the predictive risk models. The size of the bar represents the sample size of the dataset. The red portion represents the proportion of incident EC cases in the dataset, and green portion represents non-cases; darker red or green indicates an age range starting at or above 50 years, while lighter red or green indicates an age range starting below 50 years. Created with Biorender.com. Abbreviations: Prostate, Lung, Colorectal, Ovarian (PLCO) Cancer Screening Trial; National Institutes of Health – American Association of Retired Persons (NIH-AARP) Diet and Health Study; Michigan Genomics Initiative (MGI); United Kingdom Biobank (UKBB); European Prospective Investigation into Cancer (EPIC); Manchester University National Health Services (MU-NHS) Foundation Trust; Epidemiology of Endometrial Cancer Consortium (E2C2)

Most datasets used in model development included individuals at risk, with an intact uterus, who had not undergone a hysterectomy [29, 30, 32, 34, 36]. Additionally, the majority of datasets excluded individuals with pre-existing cancer, with Hüsing et al. [30] and Fortner et al. [32] including non-melanoma skin cancer [29, 30, 32, 34–37]. Additional exclusions included age restrictions [33, 34] as well as SNPs and genetic exclusion for some models [35–37]. In one case, exclusion criteria were not clearly outlined [31]. Models and datasets used in model development are summarized in Table 1 and detailed in Additional File 3, Tables S2 and S3, and visually depicted in Fig. 1.

**Table 1:** Summary of the development and validation datasets, and the performance of the included risk models

Model (Year; Region)	Development Dataset (sample size, % cases)	Validation Dataset (sample size, % cases)	AUROC (95% CI)	E/O (95% CI)	Calibration Slope
Pfeiffer (2013; US) [29]	PLCO/NIH-AARP (146679, 1.1%)	*External*: NHS (37241, 1.4%), EPIC (unspecified), UKBB (222031, 0.4%), CPRD (3094371, 0.3%)	*External*: 0.68 (0.66–0.70) 0.67 (0.63–0.70) 0.67 0.63	*External*: 1.20 (1.11–1.29)	-
Hüsing (2016; Europe) [30]	EPIC (201811, 0.4%)	*Internal*: EPIC by Hüsing, EPIC by Fortner (716, 34.5%) *External*: UKBB (222031, 0.4%) CPRD (3094371, 0.3%)	*Internal*: 0.77 (0.68–0.85) 0.63 (0.58–0.67) *External*: 0.67 0.69	*Internal*: 0.99	-
Fortner (2017; Europe) [32]	EPIC (716, 34.5%)	*Internal*: EPIC	*Internal*: 0.64 (0.60–0.68)	-	-
Hart (2020; US) [31]	PLCO (78215, 1.2%)	*Internal*: PLCO (70% train, 30% test)	*Internal*: Training (0.68–0.99) Testing (0.68–0.95)	-	-
Choi (2020; UK) [35]	UKBB (214435, 0.3%)	*Internal*: UKBB	*Internal*: 0.56 (0.54–0.58)	-	-
Fritsche (2020; US, UK) [37]	MGI (20141, 3.2%), UKBB (220896, 0.6%)	*Internal*: UKBB	*Internal*: 0.57 (0.55–0.60)	-	-
Bafligil (2022; UK) [36]	Manchester University NHS Foundation Trust (1757, 31.6%)	*Internal*: Manchester NHS, UKBB (118640, 1.4%)	*Internal*: 0.79 (0.76–0.82) 0.75 (0.73–0.76)	-	-
Shi (2023; US, Canada, Europe, Australia) [33]	E2C2 (15727, 42.4%)	*External*: NHS (68150, 1.03%), NHS genetic (11365, 1.46%), NHS II (56076, 0.5%), PLCO (39996, 1.3%), PLCO genetic (30102, 1.3%)	*External*: Epidemiologic: 0.65 (0.63–0.67) 0.69 (0.66–0.72) 0.64 (0.61–0.66) 0.63 (0.61–0.66) Epidemiologic + Genetic: 0.61 (0.57–0.66) 0.66 (0.64–0.69)	*External*: Epidemiologic: 0.55 (0.51–0.59) - 1.09 (0.98–1.22) 1.04 (0.95–1.13) Epidemiologic + Genetic: - 0.94 (0.85–1.03)	-
Kitson (2023; UK) [34]	UKBB (222031, 0.4%)	*Internal*: 100 bootstrap samples of model development dataset *External*: CPRD (3094371, 0.3%)	*Internal*: Full model: 0.74 (0.74–0.76) *External*: Any EC (Full model): 0.7 (0.69–0.70) Non-endometrioid (Full model): 0.72 (0.69–0.74)	*External*: Full model: Any EC: 1.03 (1.01–1.05)	*Internal*: Full model: 1.03 (1.01–1.06) *External*: Any EC (Full model): 1.14 (1.11–1.17) Non-endometrioid (Full model): 1.14 (1.00–1.28.00.28)

The performance of the final model of each study in their development and validation datasets. Each dataset is listed with their respective sample size and percentage of incident cancer (cases). Results of Internal and External validation are reported with 95% confidence intervals (if provided by the study). A dash (-) in the table indicates that the information is unknown or not provided

*Abbreviations*: *PLCO *Prostate, Lung, Colorectal, Ovarian Cancer Screening Trial, *NIH-AARP *National Institutes of Health – American Association of Retired Persons Diet and Health Study, *NHS *Nurses’ Health Study, *EPIC *European Prospective Investigation into Cancer, *UKBB* United Kingdom Biobank, *CPRD* Clinical Practice Research Datalink, *E2C2 *Epidemiology of Endometrial Cancer Consortium, *ANECS *Australian National Endometrial Cancer Study, *SE* Standard Error, *MGI *Michigan Genomics Initiative, *MU-NHS* Manchester University National Health Services Foundation Trust

The models were predominantly developed in datasets from Western regions (US and Europe) with limited ethnic or racial diversity. Six of the nine models were based exclusively on data from people of White or European ancestry [29, 31, 33, 35–37]. Hüsing et al. [30] and Fortner et al. [32] did not report on ethnicity, while Kitson et al. [34] had a predominantly White population (94%). Shi et al. [33] used data from the Epidemiology of Endometrial Cancer Consortium (E2C2) database, which pooled data from regions including North America, Europe, China, and Australia [42]; however, their model development was restricted to White females from the US, Alberta (Canada), Australia, and Europe.

The study design and the age of the population significantly influenced the observed incidence rate of EC, which was the primary outcome in all studies. For example, Shi et al. [33] had the highest incidence of EC (42.4%), with a case-control design and only postmenopausal participants from the E2C2 dataset. Bafligil et al. [36] used MU-NHS *(*Manchester University National Health Services) dataset [43] which also followed a case-control design with a high proportion of EC cases (31.6%). Fortner et al. [32] used a select subset of the European Prospective Investigation into Cancer (EPIC) dataset [44] where blood collection data were available; they followed a case-control design (34.5% incident EC). In contrast, the Hüsing [30] model, which considered a broader set of the EPIC dataset [44], was predominantly composed of pre- or perimenopausal participants (60%) and reported significantly lower EC incidence (0.4%). Fritsche et al. [37] used case-control data from the Michigan Genomics Initiative (MGI) [45] (3.2% incident EC) and UK Biobank (UKBB) [46], which followed a prospective design (0.6% incident EC). The remaining datasets [46, 47] were all prospective in design, with incidence varying from 0.3% to 1.2%. A detailed description of the datasets is available in Additional File 3, Table S3.

### Risk factors used in models

A diverse range of traditional epidemiological risk factors, novel biomarkers, and genetic factors were considered in model training. Some models also explored first-order interactions between risk factors [29, 30, 32–34]. The most frequently included predictors were body mass index (BMI), smoking status, and oral contraceptive (OC) use. Reproductive factors such as parity, menopausal status, age at menopause, and age at menarche were also considered, highlighting the role of reproductive history and hormonal influences in the etiology of EC. Recognizing that BMI may not fully capture obesity, several studies considered anthropomorphic variables such as weight and waist circumference [31, 34]. Hormonal exposures, like menopausal hormone therapy (MHT) (including both status and duration) and tamoxifen use, were often included [29, 30, 32–34]. Pfeiffer et al. and Hart et al. specified MHT as a combination of estrogen and progesterone therapy; Husing et al. and Kitson et al. did not indicate formulation, and Shi et al. used estrogen-only MHT. Co-morbidities, such as hypertension or diabetes, were also considered in some models [29–34]. To account for familial risk, some studies included the family history of breast, ovarian, endometrial or colorectal cancer [31, 34]. One study [32] incorporated biological data from serum biomarkers (e.g., C-reactive protein, interleukin 6, adiponectin, etc.) and evaluated those alone and in combination with epidemiological risk factors.

**Fig. 2:**
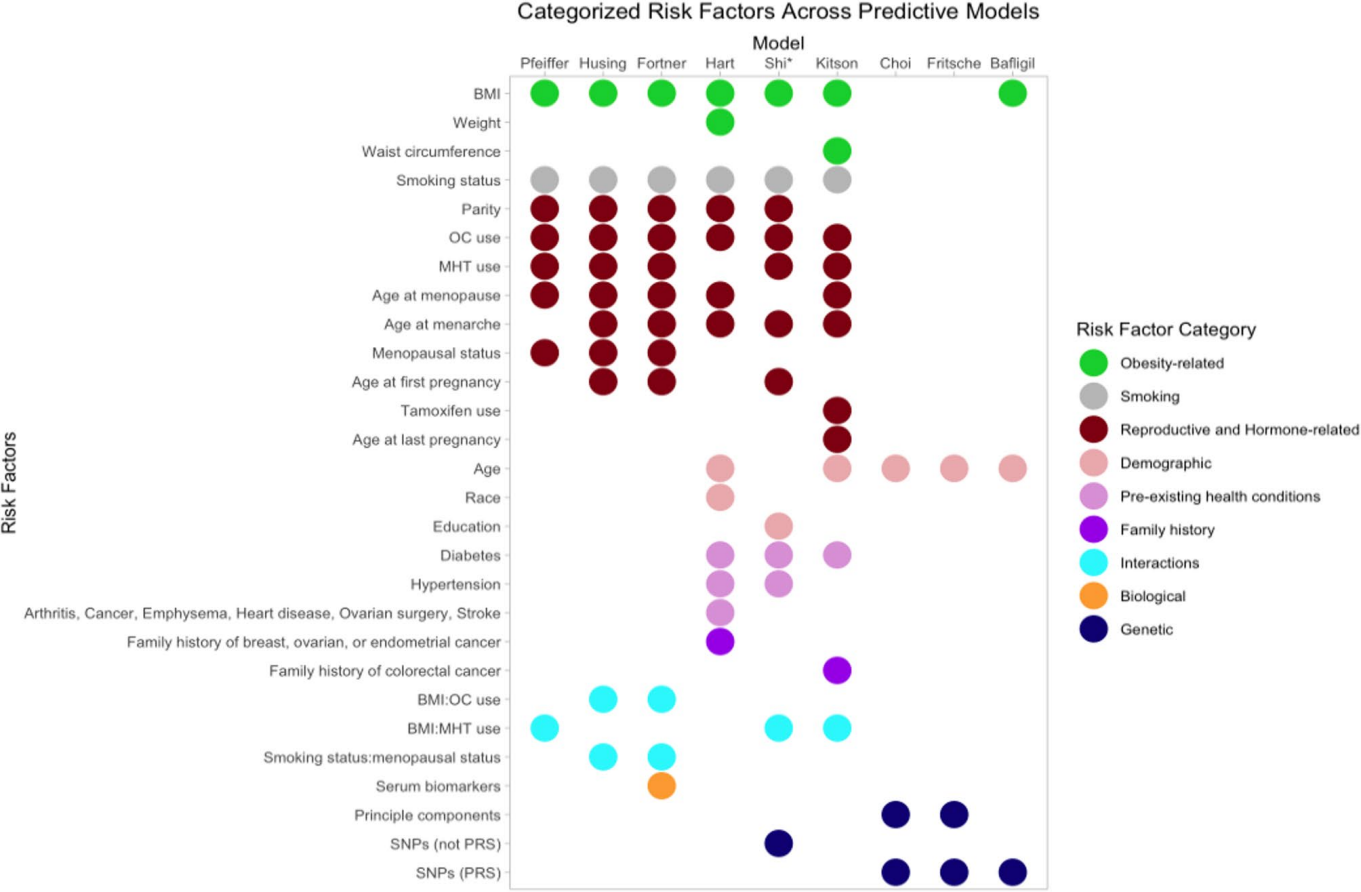
Categorized risk factors across predictive models. This visual representation facilitates easy comparison of risk factor inclusion across the various models. *Shi et al.’s model reports using SNP data directly and not through a PRS; they develop a model with epidemiological risk factors and one with epidemiological risk factors and SNPs. This figure shows Shi’s genetic model. Abbreviations: BMI (Body Mass Index); OC (Oral Contraceptive); MHT (Menopausal Hormone Therapy); SNP (Single Nucleotide Polymorphism); PRS (Polygenic Risk Score)

In contrast to models that relied on familial risk through epidemiological risk factors, some quantified genetic susceptibility by employing polygenic risk scores (PRS) derived from combining multiple SNPs [35–37]. The PRS models by Choi [35], Bafligil [36], and Fritsche [37] adjusted also for age, and Bafligil’s [36] model included BMI as well. Choi et al. [35] and Bafligil et al. [36] used 19 SNP models with 1 SNP varying between them, and Fritsche et al. [37] used a 20 SNP model, of which 15 were shared with all models. Shi et al. [33] explored the same 18 SNPs from Choi et al. [35], combining them with other epidemiological risk factors, without using a PRS model. Fig. 2; Table 2 illustrate the various risk factors across EC prediction models, while Table 3 details the various SNPs. See Additional File 4, Table S4 for all SNPs.

**Table 2:** Hazard ratios associated with final epidemiological models*

	Pfeiffer	Hüsing	Shi	Kitson
BMI (kg/m2)	1.72 (1.65–1.80) per category (< 25, 25 to < 30, 30 to < 35, 35+) *Ref: <25*	1.07 (1.06–1.09)	BMI < 18.5: 0.74 BMI ≥ 25 and < 30: 1.41 BMI ≥ 30 and < 35: 2.49 BMI ≥ 35: 5.57 *Ref: 18.5 < BMI < 25*	1.07 (1.05–1.10)
Waist Circumference (cm)				1.01 (1.00–1.02.00.02)
Smoking Status	Never smoker: 1.47 (1.22–1.78) Former smoker: 1.21 (1.00–1.47.00.47) *Ref: Current smoker*		Former smoker: 0.80 Current smoker: 0.64 *Ref: Never smoker*	Current/former smoker: 0.74 (0.65–0.86) *Ref: Never smoker*
Parity	1.21 (1.13–1.29) per category of number of children (0, 1 to 2 childre) *Ref: 3 + children*	1 child: 0.89 (0.72–1.10) 2 children: 0.80 (0.67–0.95) 3 + children: 0.6 (0.50–0.73) *Ref: Nulliparous*	1 child: 1.10 2 children: 0.91 3 children: 0.77 4 + children: 0.60 *Ref: Nulliparous*	
OC Use	< 1 year: 1.44 (1.29–1.62) *Ref: 1 + years*	0.95 (0.93–0.96)	Any OC use: 0.79 *Ref: None*	≥ 5 years: 0.73 (0.63–0.84) *Ref: Never/<5 years*
Duration of OC Use (years)			> 0 to 5: 1.05 > 5 to 10: 0.94 > 10: 0.69 *Ref: 0 years*	
Combined Hormone Use	1.15 (1.05–1.26) per category increase (never, < 10 y, 10 + y) *Ref: Never*			
Estrogen Hormone Use			> 0 to 5: 0.84 > 5 to 10: 1.42 > 10: 2.55 *Ref: 0*	
Hormone (unspecified) Use		1.07 (1.06–1.10)		Current use: 8.28 (2.06–33.26) *Ref: Never/prior use*
Tamoxifen Use				Current use: 2.91 (1.75–4.85)
Menopausal Status	Pre-menopause: 1.29 (1.01–1.63) *Ref: post-menopause*	Perimenopausal: 0.82 (0.63–1.06) Postmenopausal: 0.63 (0.48–0.84) *Ref: pre-menopause*	Only postmenopausal women were included	
Age at Menopause	1.26 (1.17–1.35) per category increase (50–54, 55+) *Ref: <50 years old*	1.07 (1.05–1.09) centered at median 50		Menopause ≥ 55 y: 1.62 (1.37–1.91) *Ref: Premenopausal/menopause < 55 y*
Age at Menarche		0.95 (0.91–0.99) centered at median 13	10–11 years: 1.04 12–13 years: 1.04 14–15: 0.92 ≥ 16: 0.89 Ref: ≤9 years old	0.90 (0.86–0.95)
Age at First Pregnancy		0.96 (0.95–0.98) centered at median 24	20 to < 25: 0.96 25 to < 30: 0.85 30 to < 35: 0.83 ≥ 35: 0.84 *Ref: <20 years*	
Age at Last Pregnancy				0.98 (0.98–0.99)
Age				1.06 (1.05–1.07)
Education			Some college or equivalent: 0.97 College or above: 0.96 Ref: High school or below	
Diabetes			1.39	1.43 (1.11–1.84)
Hypertension			1.22	
Family history of colorectal cancer				1.16 (0.96–1.41)
BMI: OC use		OC use (ever) x BMI < 25 kg/m2: 0.96 (0.79–1.16) OC use (ever) x BMI ≥ 25 to ≤ 30: 0.74 (0.48–1.14) OC use (ever) x BMI ≥ 30: 0.92 (0.57–1.48) *Ref: No OC use*		
BMI x Combined Hormone Use	1.61 (1.43–1.81) *Ref: CHT use* x *BMI < 25*			
BMI x Any Hormone Use			Any MHT use x BMI 25 to < 30: 0.87 Any MHT use x BMI 30 to < 35 kg/m2: 0.64 Any MHT use x BMI ≥ 35 kg/m2: 0.60 *Ref: No MHT use*	0.94 (0.89–0.99)
Smoking status x menopausal status		Premenopausal, former smoker: 0.78 (0.56–1.09) Premenopausal, current smoker: 1.10 (0.80–1.51) Premenopausal, unknown status: 1.45 (0.61–3.48) Perimenopausal, former smoker: 0.73 (0.32–1.68) Perimenopausal, current smoker: 1.00 (0.44–2.27) Perimenopausal, unknown status: 1.33 (0.17–10.23) Postmenopausal, former smoker: 0.77 (0.38–1.56) Postmenopausal, current smoker: 0.62 (0.30–1.30) Postmenopausal, unknown status: 0.44 (0.04–4.69) *Ref: Never smoker*		

Outlines the variables in each risk model along with associated hazard ratio. Note that some risk models used different terms for the same risk variable, those were harmonized to the extent possible

*Abbreviations*: *BMI *Body Mass Index, *OC *Oral Contraceptive, *MHT *Menopausal Hormone Therapy

*The Hart et al. was a machine learning model and did not provide hazard ratios

**Table 3:** Odds ratios associated with final PRS models only*

Model	Bafligil	Shi
rs113998067	1.24	1.23 (1.14–1.32)
rs148261157	1.26	1.26 (1.16–1.36)
rs1740828	0.87	1.15 (1.11–1.19)
rs2747716	0.90	1.10 (1.07–1.14)
rs4733613	0.85	1.18 (1.13–1.24)
rs139584729	0.71	1.40 (1.25, 1.58)
rs35286446	1.08	-
rs1679014	0.84	1.18 (1.12, 1.25)
rs10835920	1.09	1.09 (1.06, 1.13)
rs9668337	1.11	1.11 (1.08–1.15)
rs3184504	1.10	1.10 (1.07–1.14)
rs10850382	1.10	1.10 (1.07–1.14)
rs7981863	0.86	1.16 (1.12–1.20)
rs2498796	-	1.07 (1.03–1.11)
rs937213	1.09	1.09 (1.06–1.13)
rs17601876	1.12	1.12 (1.09–1.16)
rs1129506	0.91	1.10 (1.06–1.13)
rs11263761	1.15	1.15 (1.12–1.19)
rs882380	1.10	1.10 (1.06–1.13)

Contains all hazard ratios associated with SNPs of the final SNP-based model. All SNPs are listed as rsIDs. Dashes (-) indicate that the row wise SNP is not included in that model

*Note that Choi et al. and Fritsche et al. did not provide odds ratios in their main manuscript or supplementary material

### Methodologies for risk computation

Six predictive models calculated absolute risk (AR) [29–34], which provides a personalized estimate of the likelihood of developing EC over a specific period (usually 5 or 10 years). These models integrate individual risk factors with baseline risk derived from a comparable population reference dataset and account for competing risks. Models used variations of the cause-specific hazard function, which calculates the instantaneous risk of EC at a given specific time, conditional on the event not having occurred yet. The Hüsing [30] model incorporated country-specific incidence adjustments to improve its predictive accuracy by tailoring it to different European populations.

To compute AR, models need to first estimate relative risk (RR), which measures how the presence or absence of specific risk factors affects the likelihood of developing EC [38]. While RR is useful for identifying risk factors, it does not offer a personalized risk measure. Cox proportional hazards regression was used by several models [29, 30, 34, 35], while others used logistic regression [32, 36, 37]. Cox regression enables the modelling of time-to-event outcomes and accounts for censored data due to loss to follow-up [38]. Logistic regression predicts the occurrence of an event at a specific time point. While considered simple, logistic regression cannot account for the censoring due to the loss of follow-up [48]. Hart et al. [31] evaluated seven ML algorithms and compared their performance, but did not publicly publish any model codes or parameters, making reproducibility difficult.

To refine their models, many studies used backward elimination to remove the least significant variables iteratively [29, 30, 32, 34]. Some used penalized likelihood and shrinkage approaches for variable selection and to mitigate overfitting [33, 37]. In genetic models, Fritsche et al. [37] used three methods to develop distinct PRSs for EC, with their final model using pruning and thresholding to optimize SNP selection. Bafligil et al. [36] created PRSs by assigning log odds ratios to each SNP and summing the product of the ratios with corresponding weights to obtain a standardized risk score. Similarly, Choi et al. [35] used regression coefficients from prior literature for each SNP to compute PRS scores.

### Performance of risk models

Model performance was assessed by considering both discrimination and calibration [49]. Discrimination reflects how well a model distinguishes between those who develop EC and those who do not, typically measured using the AUROC or C-statistic [50]. Conversely, calibration measures how closely predicted risks align with observed outcomes, often evaluated using the expected-to-observed (E/O) ratio or calibration slopes [50]. Calibration requires the assessment of AR. Many models were only internally validated, with only five externally validated in datasets not used for training [29, 30, 33, 34, 36]. Most EC risk models demonstrated moderate to good discrimination, with AUROCs ranging between 0.60 and 0.79.

The Pfeiffer et al. [29] model showed moderate discriminative ability, with an AUROC of 0.68 in the Nurses’ Health Study (NHS), which included White participants aged 30–55, a cohort composition different from the one the model was trained on [51]. The model overestimated risk, with an E/O ratio of 1.20, which worsened during validation in the UKBB [46] and Clinical Practice Research Datalink (CPRD) datasets [52]. The Hüsing [30] model had better discrimination and calibration with AUROC of 0.77 (95% CI: 0.68–0.85) and an E/O ratio of 0.99 in internal validation. When validated externally in UKBB and CPRD cohorts, the Husing et al. model resulted in discrimination similar to Pfeiffer et al. [29] but continued to be better calibrated. Fortner et al. [32] attempted to improve upon Hüsing’s [30] model by introducing biological markers, but the improvement in discrimination was modest. Hart et al. [31] used ML approaches that showed promising internal validation results, particularly for Random Forest (AUROC 0.95) and Neural Networks (AUROC 0.88). The Shi et al. [33] models validated on the NHS [51], NHS II [51], and the Prostate, Lung, Colorectal, and Ovarian Cancer Screening Trial (PLCO) [47], all US datasets of non-Hispanic White, postmenopausal females and showed moderate discrimination, with AUROCs between 0.64 and 0.69. Adding SNP data resulted in only modest improvements in discrimination in the PLCO [47] dataset and did not improve performance in the NHS [51] datasets. Shi’s [33] epidemiologic model was well-calibrated in NHS II [51] and PLCO [47], but poorly calibrated in the NHS [51] dataset. The Kitson et al. [34] model was validated only on the CPRD [52] data and showed good overall performance, with a C-statistic of 0.70 (95% CI: 0.69–0.70), a calibration slope of 1.14 (95% CI: 1.11–1.17), and an E/O ratio of 1.03 (95% CI: 1.01–1.05).

The PRS-based models [35–37] generally demonstrated poor to moderate discriminative abilities in validation. The Choi [35] and Fritsche [37] models exhibited AUROCs below 0.60 when internally validated in the UKBB [46], indicating poor discrimination. In contrast, the Bafligil [36] model, which included both PRS and BMI, performed better with an AUROC of 0.79 during internal validation and 0.75 in external validation on the UKBB dataset. Fritsche [37] accounted for correlation among SNPs through linkage disequilibrium pruning and penalized regression, whereas Balfigil [36] and Choi [35] assumed independence of SNPs. Both the Kitson et al. [34] and Bafligil et al. [36] models (PRS only) were also considered for predicting overall and non-endometrioid EC risk, demonstrating comparable results for both outcomes.

### Quality assessment of models

Evaluation of quality of reporting as per the TRIPOD checklist [41] demonstrated that among epidemiological models, Hart et al. [31] had the lowest level of reporting quality, especially in the methods section. Key shortcomings included insufficient reporting of risk factor handling, model parameters including tuning and hyperparameters, and feature selection. Moreover, the final ML model was not provided, and therefore their results would be difficult to reproduce. The Pfeiffer [29], Shi [33], and Kitson [34] models followed high reporting quality, with Kitson et al. [34] performing highest across all sections. The Husing [30] model was of medium overall quality, as well as Fortner et al. [32], who did not share their codes and missed several key points in their title and introduction. For PRS-based models, Bafligil et al. [36] had high overall quality. Choi et al. [35] as medium, while Fritsche et al. [37] were considered of poor reporting quality due to their vague abstracts and titles lacking clear descriptions of the models and outcomes. Fritsche et al. [37] also failed to provide necessary details on model parameters and omitted important evaluation metrics from the results section, further contributing to poor quality. Full details of the quality of reporting assessment are presented in Additional File 5, Table S5.

## Discussion

This study provides a comprehensive review of predictive models developed to estimate EC risk in asymptomatic individuals. Our evaluation highlights key trends in model development, the strengths and limitations of various approaches, and how these can impact the clinical applicability of existing models.

Predictive models for EC have evolved from those using only traditional epidemiological risk factors to more complex models incorporating genetic and biomarker data. Epidemiologic-based models such as those developed by Pfeiffer et al. [29], Hüsing et al. [30], Hart et al. [31], Fortner et al. [32], Shi et al. [33], and Kitson et al. [34] frequently used variables including BMI, smoking, OC use, MHT, parity, and age, which are well-established risk factors consistently associated with EC risk [53–56]. Most models demonstrated moderate discrimination on external validation ranging from AUROC 0.63–0.7 and varied calibration. Models did not age-adjust AUROC. This poses a challenge because age is a strong determinant of cancer risk; failure to adjust for age can lead to inflated estimates of discrimination. Biological and genetic markers, including serum biomarkers and SNPs, have shown limited effectiveness when used in isolation, providing only modest improvements to traditional epidemiological models in predicting EC risk. Models solely based on PRS, or biological data generally had poor discrimination performance. The predictive power of these biological and genetic markers is enhanced when used in conjunction with other clinical and epidemiological data rather than in isolation. In the Shi [33] model, the combination of epidemiologic risk factors and SNPs resulted in an AUROC improvement of 3% compared to their traditional epidemiologic model. Bafligil et al. [36] saw a 2% increase in AUROC with the addition of PRS in their BMI and age model. Similarly, Fortner et al.’s [32] discrimination improved by 2% and 1.7% with the addition of all biomarkers and selected biomarkers, respectively. Epidemiologic-only models are immediately applicable in many clinical and population settings given their reliance on routinely collected data. In contrast, biomarker- and SNP-based models may currently require higher resource settings, but their clinical utility is likely to expand as costs decline and technologies become more widely accessible, broadening the contexts in which such models could be applied for risk stratification and screening.

While some models, like Hart et al. [31], attempted to leverage ML algorithms, the lack of transparency in their reporting of models and model parameters will hinder their external validation and applicability. Improved adherence to reporting guidelines, as seen in several studies [29, 33, 34, 36], would significantly enhance the credibility and utility of the models by supporting future validation and clinical application.

One of the primary challenges identified in this review is the limited generalizability of models, predominantly developed using datasets from Western populations, particularly women of White ethnicity or European ancestry. While epidemiological risk models have been validated in several datasets, many SNP-based models lack external validation, suggesting likely overoptimism in their reported results. Moreover, assuming independence of SNPs is unlikely to hold in the presence of linkage disequilibrium and may inflate performance estimates, highlighting a limitation when comparing models that rely on different assumptions. The absence of diverse ethnic groups in the data used for model training may limit their ability to accurately predict risk for women from different racial backgrounds, raising concerns about their potential to exacerbate the health disparities in EC outcomes [57]. EC is known to evolve from different pathways [58, 59]: the estrogen-dependent, often linked to obesity, with hyperplasia as a precursor, resulting in low-grade endometrioid EC and non-estrogen-dependent, typically more aggressive non-endometrioid and high-grade EC [60–62]. It is generally understood that non-endometrioid and high-grade EC arise in older populations of non-White ethnicity and are less likely to have obesity and hyperplasia as precursors [63]. Non-endometrioid EC diagnoses, particularly serous, may be driving worse outcomes and inequity [64–66]. Although Kitson et al. [34] and Balgifil et al. [36] investigated EC risk prediction between non-endometrioid EC and all types of EC, the number of non-endometrioid cases in the datasets used, and the predominantly White ethnic backgrounds of participants may have resulted in limited power to detect differences. Moreover, models were not trained to discriminate between the types of EC. Incorporating additional outcomes like endometrial hyperplasia, endometrial intraepithelial neoplasia, and specific subtypes of EC is essential because these conditions represent precursors or distinct pathways in the development of EC [67]. Training models to detect these outcomes would enable more nuanced risk prediction, capturing the full spectrum of disease progression. This can improve the models’ ability to stratify patients for tailored prevention strategies, early detection, and intervention, particularly in populations at varying stages of disease or with differing risk profiles.

Among risk factors considered in modeling, BMI stands out as a particularly strong predictor. EC is highly influenced by obesity, as excess adipose tissue leads to increased estrogen production, which promotes endometrial proliferation [68]. Many studies suggest a dose-response, with EC risk increasing faster than linearly with increased obesity. Furthermore, an association with the timing of the onset of obesity and the duration of exposure with increased endometrial risk has been noted. Smoking is associated with a decreased risk of EC, particularly endometrioid subtypes, likely due to its anti-estrogenic effects [54]. However, it also poses an increased risk for many other cancers and health issues, complicating its clinical implications. This dual effect makes smoking a challenging factor to interpret in EC risk prediction, and it is typically included in models as part of a broader risk profile. Oral contraceptive use is consistently shown to reduce the risk of EC by suppressing endometrial proliferation [60, 65, 69]. Most models incorporate OC and MHT use as a protective factor, but the degree of risk reduction depends on the duration and recency of OC use. Moreover, the type of hormone used (estrogen, progestogen or combination) in OC and MHT was not fully captured by models. In recent years, the use of OC has been declining [70], making way for hormone-based intrauterine devices (IUD) such as those containing the progestogen levonorgestrel, which play a significant role in reducing EC risk [71]. Recent decades have seen an increased use of these devices for contraception and to manage abnormal uterine bleeding [72]. MHT is another important factor for risk, with estrogen-only MHT linked to increased EC risk due to unopposed estrogen effects, particularly in women with an intact uterus [73]. Progestogen-only or combined MHT (estrogen and progestin) mitigates this risk by counteracting estrogen’s effect on the endometrium [73]. Models like Pfeiffer et al. [29] and Hüsing et al. [30] explicitly include MHT in their risk calculations. However, the variations in formulations, dosages, and durations of MHT use are often oversimplified in these models, limiting their precision. A major limitation of existing predictive models is that they are constrained by the risk factors included in the dataset they were trained on, which may only comprehensively represent some relevant exposures related to EC. The scope of risk factors is often narrow and may overlook important exposures, including environmental exposures, such as pollutants and endocrine-disrupting chemicals, which are increasingly recognized for their role in carcinogenesis [74].

Most models reviewed focused on postmenopausal women. This aligns with the increased risk of EC in that population, but it also means that premenopausal individuals are underrepresented in most models. As EC rates are rising in younger populations, particularly those with risk factors such as obesity [9, 75, 76]. Perimenopause could provide a window of opportunity for prevention; the ability to predict risk and intervene before cancer arises [75, 77], can reduce the incidence of EC and improve overall health outcomes. The exclusion of reproductive-age women in model development may delay early detection and preventive interventions. Similarly, focusing on complete cases [33] and excluding individuals based on missing follow-up data [30] can introduce bias. The choice between using time-to-event vs. binary outcomes in modeling EC incidence is critical. Using the Cox model allows researchers to adjust for censored outcomes and loss of follow-up and account for time-varying exposures [78, 79]. In contrast, a binary outcome modelled with logistic regression is more straightforward but ignores censoring and may introduce bias [80, 81]. There is also significant potential for enhancing risk modeling through machine learning, which could account for non-linear relationships within the data, offering greater flexibility in model design. However, machine learning models would still require extensive external validation to mitigate the risk of overfitting. This adaptability enables incorporating a broader range of complex and interrelated risk factors that traditional statistical methods may struggle to handle.

Many existing models are static, computed at a single point in time, which limits their ability to incorporate factors that can modify risk, such as hysterectomies, IUD use, and weight loss. For example, some models adjusted for hysterectomy rates based on average obtained from the Surveillance, Epidemiology, and End Results (SEER) [82]. Calibration is essential for ensuring that predictions are accurate and clinically meaningful. Reliable calibration relies on a robust baseline risk encompassing various relevant factors, including demographic, lifestyle, and medical history variables. Integrating SES in baseline risk may enhance the models’ relevance and improve their applicability in diverse populations. A well-calibrated model enhances confidence in its predictions and subsequently would have a greater impact in guiding clinical decision-making.

### Conclusion

While EC satisfies several of the World Health Organization (WHO) criteria for screening [21], such as being a well-understood disease with effective treatments, the feasibility of population-wide screening remains a challenge. A targeted, risk-based screening approach may be appropriate for EC by focusing on high-risk populations where screening and prevention efforts would be most beneficial and cost-effective. Personalized risk prediction has been identified as a top priority for patients and clinicians alike [83]. The predictive models for EC risk reviewed here offer valuable insights into disease risk estimation but face limitations in generalizability, transparency, and external validation, particularly in bridging the racial equity gap. Moving forward, predictive modelling must prioritize inclusion, transparency, and validation to ensure clinical applicability. By addressing these gaps, future models can better support personalized risk assessment and early detection strategies for EC, ultimately improving patient outcomes.

## Supplementary Information

Additional File 1. PRISMA Checklist.

Additional File 2. Details of search strategies and exclusions. 

Additional File 3. The PRISMA study selection flow diagram.

Additional File 4. Table S1 (Reviewed Studies), Table S2 (Models), Table S3 (datasets). 

Additional File 5. Table S4 (All SNP Models).

Additional File 6. Table S5 (Quality Assessment of Risk Models based on TRIPOD Guidelines and Checklist).

## Data Availability

Data supporting the conclusions of this article are all included in the article and additional files. Original data were obtained from papers that are publicly accessible from the PubMed and Ovid MEDLINE databases. The search strategy used to collect these data can be replicated by utilizing the search strings provided in Additional File 2. Since these databases are publicly available, the data are not stored in a separate repository. Using the provided search queries, readers can access the original data directly through the PubMed and Ovid MEDLINE platforms.

## Abbreviations

EC: Endometrial Cancer
SES: Socio-economic Status
PRISMA: Preferred Reporting Items for Systematic Reviews and Meta-Analyses
MeSH: Medical Search Heading
AUROC: Area Under the Receiver Operating Curve
E/O Ratio: Expected/Observed Ratio
TRIPOD: Transparent Reporting of a Multivariable Prediction Model
US: United States
ML: Machine learning
SNP: Single Nucleotide Polymorphism
UK: United Kingdom
E2C2: Epidemiology of Endometrial Cancer Consortium
EPIC: European Prospective Investigation into Cancer
MU-NHS: Manchester University National Health Services
MGI: Michigan Genomics Initiative
UKBB: United Kingdom Biobank
BMI: Body Mass Index
OC: Oral Contraceptive
MHT: Menopausal Hormone Therapy
AR: Absolute Risk
RR: Relative Risk
PRS: Polygenic Risk Score
NHS or NHSII: Nurses’ Health Study
CPRD: Clinical Practice Research Datalink
PLCO: Prostate, Lung, Colorectal, and Ovarian Cancer Screening Trial
IUD: Intrauterine Device
SEER: Surveillance, Epidemiology, and End Results
WHO: World Health Organization
