# Supplementary material for: Insights and limitations of endometrial cancer risk prediction models for clinical applicability: a systematic review
Source: BMC Cancer. 2025 Nov 19;25:1787. doi: 10.1186/s12885-025-15200-x (PMC12628995; doi:10.1186/s12885-025-15200-x)
Supplement: Supplementary file 2 — Additional File 2. Details of search strategies and exclusions. [file 12885_2025_15200_MOESM2_ESM.docx]

**Additional File 2:** Details of Search Strategies and Exclusions.

**MEDLINE (Ovid) search strategy**

**Search string:**

| # | Search |
| --- | --- |
| 1 | exp Endometrial Neoplasms/ or endometrial cancer.mp or endometrial neoplasm*.mp or endometrial carcinoma*.mp. or uterine cancer.mp. or uterine neoplasm*.mp. or uterine carcinoma*.mp. |
| 2 | (risk or risk factor* or risk score* or population* at risk or risk factor score* or risk assessment* or risk analys* or incidence or asymptomatic or presymptomatic or pre-symptomatic).mp. [mp=title, book title, abstract, original title, name of substance word, subject heading word, floating sub-heading word, keyword heading word, organism supplementary concept word, protocol supplementary concept word, rare disease supplementary concept word, unique identifier, synonyms, population supplementary concept word, anatomy supplementary concept word] |
| 3 | (predict* or model*).mp. |
| 4 | (prognos* or recurr* or (preoperati* or pre-operati*)).ti. |
| 5 | 1 and 2 and 3 |
| 6 | 5 not 4 |
| 7 | limit 6 to (english language and yr="2000 -Current") |

<https://access.ovid.com/custom/redirector/index.html?dest=https://go.openathens.net/redirector/ubc.ca?url=http://ovidsp.ovid.com/ovidweb.cgi?T=JS&NEWS=N&PAGE=main&SHAREDSEARCHID=7PSTpaBFQ06GR6PCuSHo8kJZM6EiBj11jRoJNZpBxGOEZMLL3HL0lav5N5uFHrIRQ>

**MEDLINE results:**

- Total results: 3637 papers
- Full text reviewed: 25 papers
- Exclusions from full text reviews and reason
  - Diagnostic models (4 papers):
    - Kuai D, Tang Q, Tian W, et al. Rapid identification of endometrial hyperplasia and endometrial endometrioid cancer in young women. *Discov Onc*. 2023;14:121. doi:10.1007/s12672-023-00736-w.
    - Wang J, Song P, Zhang M, Liu W, Zeng X, Chen N, Wang M. A prediction model based on deep learning and radiomics features of DWI for the assessment of microsatellite instability in endometrial cancer. *Cancer Med*. 2024;13(16). doi:10.1002/cam4.70046.
    - Heremans R, Wynants L, Valentin L, Leone FPG, Pascual MA, Fruscio R, Van den Bosch T. Estimating risk of endometrial malignancy and other intracavitary uterine pathology in women without abnormal uterine bleeding using ieta-1 multinomial regression model: validation study. *Ultrasound Obstet Gynecol*. 2024;63(4):556-563. doi:10.1002/uog.27530.
    - Vitale SG, Angioni S, D'Alterio MN, Ronsini C, Saponara S, De Franciscis P, Riemma G. Risk of endometrial malignancy in women treated for breast cancer: the BLUSH prediction model - evidence from a comprehensive multicentric retrospective cohort study*. Climacteric.* 2024;27(5):482-488. doi:10.1080/13697137.2024.2376189.
  - Prognostic models (5 papers):
    - Bruno V, Betti M, D’Ambrosio L, et al. Machine learning endometrial cancer risk prediction model: integrating guidelines of the European Society for Medical Oncology with the tumor immune framework. *Int J Gynecol Cancer.* 2023;33:1708–1714. doi:10.1136/ijgc-2023-004671.
    - Sang Q, Yang L, Zhao H, et al. Risk prediction model of uterine corpus endometrial carcinoma based on immune-related genes. *BMC Women's Health.* 2024;24:429. doi:10.1186/s12905-024-03237-2.
    - Beavis AL, Blechter B, Najjar O, Fader AN, Katebi Kashi P, Rositch AF. Identifying women 45 years and younger at elevated risk for endometrial hyperplasia or cancer. *Gynecol Oncol*. 2023;174:98–105. doi:10.1016/j.ygyno.2023.04.019.
    - Kolehmainen AM, Pasanen AM, Koivisto-Korander RL, Butzow RC, Loukovaara MJ. Molecular characterization in the prediction of disease extent in endometrial carcinoma. *Eur J Obstet Gynecol Reprod Biol.*  2021256doi:10.1016/j.ejogrb.2020.10.031
    - Zhou L, Meng Z, Wu Y, Zhu H, Wang X. Prediction of endometrial carcinogenesis probability while diagnosed as atypical endometrial hyperplasia: a new risk model based on age, CA199 and CA125 assay. *Eur J Obstet Gynecol Reprod Biol.* 2014183doi:10.1016/j.ejogrb.2014.10.006
  - Not multivariable models (3 papers):
    - Hartman TJ, McCullough ML, Hodge JM, Gaudet MM, Wang Y, Gapstur SM. Dietary energy density, glycemic load, glycemic index, and risk for endometrial cancer in the CPS - II Nutrition Cohort. *Cancer Epidemiol Biomarkers Prev* . 2018;27(1):113 - 115. doi:10.1158/1055 - 9965.EPI - 17 – 0964
    - Yang HP, Wentzensen N, Trabert B, et al. Endometrial cancer risk factors by 2 main histologic subtypes: the NIH-AARP Diet and Health Study. *Am J Epidemiol.* 2013;177(2):142-51. doi:10.1093/aje/kws200
    - Rota M, Rumi F, Bagnardi V, et al. Modelling body mass index and endometrial cancer risk in a pooled-analysis of three case-control studies. *BJOG*. 2016;123(2):285-92. doi:10.1111/1471-0528.13717
  - Irrelevant outcome (2 papers)
    - Wang F, Pang R, Shi S, Zhang Y. Construction and validation of a clinical risk model based on machine learning for screening characteristic factors of lymphovascular space invasion in endometrial cancer. *Sci. rep.* 2024;14(1):12624. doi:10.1038/s41598-024-63436-7
    - Zhang YD, Hurson AN, Zhang H, et al. Assessment of polygenic architecture and risk prediction based on common variants across fourteen cancers. *Nat Commun*. 2020;11(1):3353. doi:10.1038/s41467-020-16483-3
  - Assessed symptomatic individuals (3 papers):
    - Ruan H, Chen S, Li J, et al. Development and Validation of a Nomogram Prediction Model for Endometrial Malignancy in Patients with Abnormal Uterine Bleeding. Yonsei Med J. . 2023;64(3):197-203. doi:10.3349/ymj.2022.0239 <https://doi.org/10.3349/ymj.2022.0239>
    - Burbos N, Musonda P, Duncan TJ, Crocker SG, Morris EP, Nieto JJ. Estimating the risk of endometrial cancer in symptomatic postmenopausal women: a novel clinical prediction model based on patients' characteristics. *Int J Gynecol Cancer*. 2011;21(3):500-6. doi:10.1097/IGC.0b013e31820c4cd6
    - Burbos N, Musonda P, Giarenis I, et al. Predicting the risk of endometrial cancer in postmenopausal women presenting with vaginal bleeding: the Norwich DEFAB risk assessment tool. *Br J Cancer.* 2010;102(8):1201-6. doi:10.1038/sj.bjc.6605620
- Final inclusion = 8 papers

_____________________________________________________________________________

**PubMed search strategy**

**Search String:**

((("endometrial cancer" OR "endometrial neoplasm*" OR "endometrial carcinoma*" OR "uterine cancer" OR "uterine neoplasm*" OR "uterine carcinoma*") AND (risk OR "risk factor*" OR "risk score*" OR "population* at risk" OR "risk factor score*" OR “risk assessment*” OR “risk analys*” OR incidence OR asymptomatic OR presymptomatic OR pre-symptomatic) AND (predict* OR model*)) NOT prognos* NOT recurr* NOT (preoperati* OR pre-operati*))

Filtered for papers written in English published on or after January 1, 2000.

Language: English

**PUBMED results:**

- Total results (titles/abstracts reviewed): 2633 papers
- Full text reviewed: 13 papers
- Exclusions from full text reviews and reason
  - Diagnostic models (2 papers)
    - Erdemoglu E, Serel TA, Karacan E, Köksal OK, Turan İ, Öztürk V, Bozkurt KK. Artificial intelligence for prediction of endometrial intraepithelial neoplasia and endometrial cancer risks in pre- and postmenopausal women. *AJOG Global Reports*. 2023;3(1):100154. doi:10.1016/j.xagr.2022.100154.
    - Hutt S, Mihaies D, Karteris E, Michael A, Payne AM, Chatterjee J. Statistical meta-analysis of risk factors for endometrial cancer and development of a risk prediction model using an artificial neural network algorithm. *Cancers*. 2021;13(15):3689. doi:10.3390/cancers13153689.
  - Prognostic models (1 paper)
    - Jayawickrama IU, Abeysena C. Development of a risk prediction model for endometrial carcinoma among postmenopausal women in the Western Province of Sri Lanka. *Ceylon Med J*. 2022;67(4):169-176. doi:10.4038/cmj.v67i4.9746.
  - Assessed symptomatic individuals (1 paper)
    - Giannella L, Cerami LB, Setti T, Bergamini E, Boselli F. Prediction of Endometrial Hyperplasia and Cancer among Premenopausal Women with Abnormal Uterine Bleeding. *Biomed Res Int.* 20192019doi:10.1155/2019/8598152
  - Irrelevant outcome (1 paper)
    - Zhang YD, Hurson AN, Zhang H, et al. Assessment of polygenic architecture and risk prediction based on common variants across fourteen cancers. *Nat Commun*. 2020;11(1):3353. doi:10.1038/s41467-020-16483-3
- Final inclusion = 8 papers

_____________________________________________________________________________

**Duplicates among the 2 databases: 8 papers**

- Pfeiffer RM, Park Y, Kreimer AR, et al. Risk prediction for breast, endometrial, and ovarian cancer in white women aged 50 y or older: derivation and validation from population-based cohort studies. *PLoS Med*. 2013;10(7):e1001492. doi:10.1371/journal.pmed.1001492
- Hüsing A, Dossus L, Ferrari P, et al. An epidemiological model for prediction of endometrial cancer risk in Europe. *Eur J Epidemiol*. 2016;31(1):51-60. doi:10.1007/s10654-015-0030-9
- Hart GR, Yan V, Huang GS, et al. Population-based screening for endometrial cancer: Human vs. Machine intelligence. *Front Artif Intell*. 2020;3:539879. doi:10.3389/frai.2020.539879
- Fortner RT, Hüsing A, Kühn T, et al. Endometrial cancer risk prediction including serum-based biomarkers: results from the EPIC cohort. *Int J Cancer*. 2017;140(6):1317-1323. doi:10.1002/ijc.30560
- Shi J, Kraft P, Rosner BA, et al. Risk prediction models for endometrial cancer: development and validation in an international consortium. *J Natl Cancer Inst*. 2023;115(5):552-559. doi:10.1093/jnci/djad014
- Kitson SJ, Crosbie EJ, Evans DG, et al. Predicting risk of endometrial cancer in asymptomatic women (PRECISION): Model development and external validation. *BJOG*. Published online December 10, 2023. doi:10.1111/1471-0528.17729
- Choi J, Jia G, Wen W, Long J, Zheng W. Evaluating polygenic risk scores in assessing risk of nine solid and hematologic cancers in European descendants. *Int J Cancer*. 2020;147(12):3416-3423. doi:10.1002/ijc.33176
- Bafligil C, Thompson DJ, Lophatananon A, et al. Development and evaluation of polygenic risk scores for prediction of endometrial cancer risk in European women. *Genet Med*. 2022;24(9):1847-1856. doi:10.1016/j.gim.2022.05.014
  - Found through manual search of references:

Fritsche LG, Patil S, Beesley LJ, et al. Cancer PRSweb: An online repository with polygenic risk scores for major cancer traits and their evaluation in two independent biobanks. *Am J Hum Genet*. 2020;107(5):815-836. doi:10.1016/j.ajhg.2020.08.025

_____________________________________________________________________________

**Manual Search (1 paper)**

- Fritsche LG, Patil S, Beesley LJ, et al. Cancer PRSweb: An online repository with polygenic risk scores for major cancer traits and their evaluation in two independent biobanks. *Am J Hum Genet*. 2020;107(5):815-836. doi:10.1016/j.ajhg.2020.08.025

_____________________________________________________________________________

**Final tally: 9 papers**
