# Supplementary material for: Insights and limitations of endometrial cancer risk prediction models for clinical applicability: a systematic review
Source: BMC Cancer. 2025 Nov 19;25:1787. doi: 10.1186/s12885-025-15200-x (PMC12628995; doi:10.1186/s12885-025-15200-x)
Supplement: Supplementary file 3 — Additional File 3. The PRISMA study selection flow diagram. [file 12885_2025_15200_MOESM3_ESM.docx]

**Additional Fig 1.** The PRISMA Study Selection Flow Diagram.


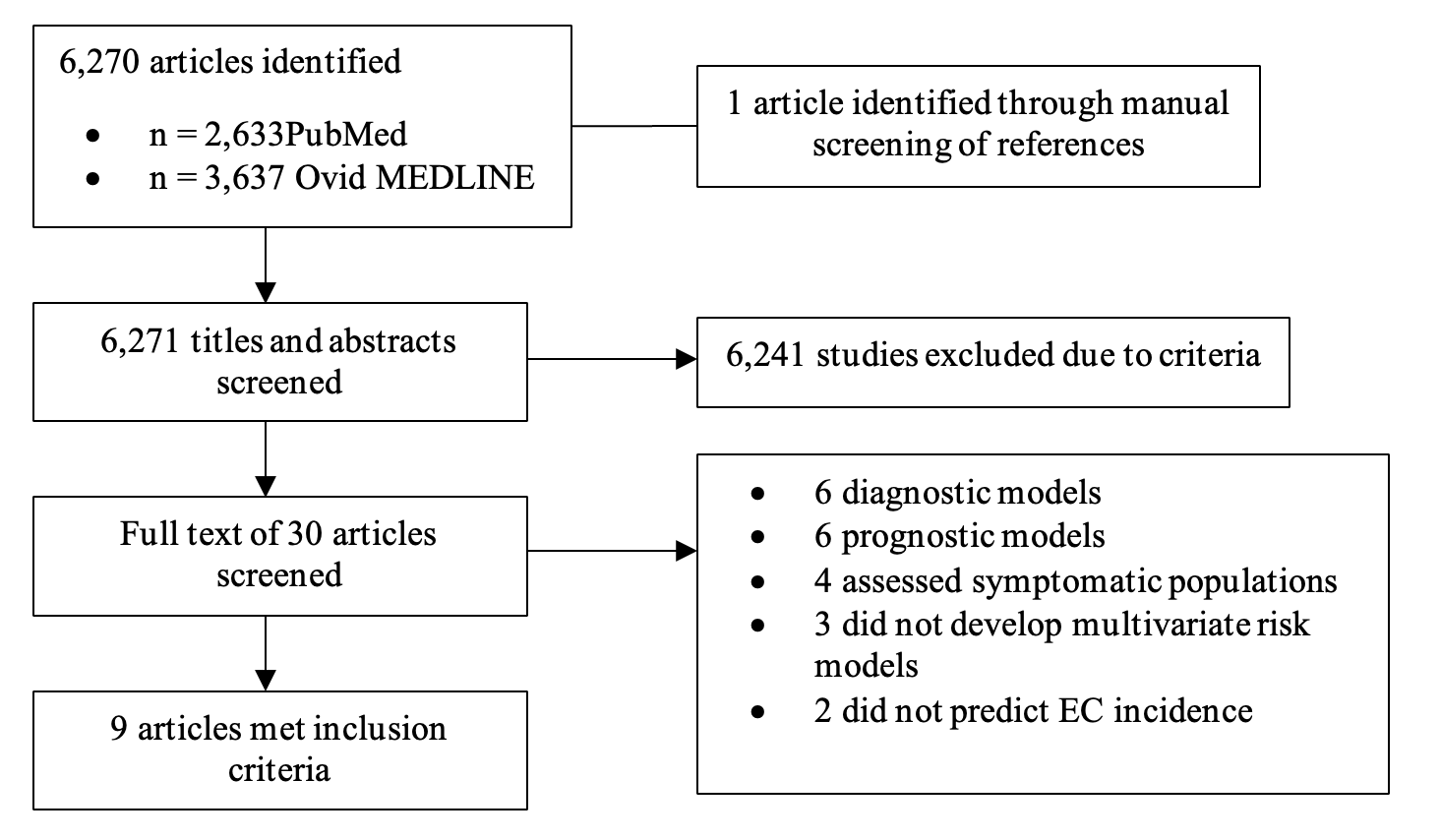


*This figure provides the study selection flow in accordance with PRISMA requirements. The figure outlines the initial extraction numbers from each search engine, and the stepwise exclusion of the articles and why they were excluded.*
