# Supplementary material for: Insights and limitations of endometrial cancer risk prediction models for clinical applicability: a systematic review
Source: BMC Cancer. 2025 Nov 19;25:1787. doi: 10.1186/s12885-025-15200-x (PMC12628995; doi:10.1186/s12885-025-15200-x)
Supplement: Supplementary file 6 — Additional File 6. Table S5 (Quality Assessment of Risk Models based on TRIPOD Guidelines and Checklist). [file 12885_2025_15200_MOESM6_ESM.docx]

**Additional File 5, Table S5**. Quality assessment of risk models based on TRIPOD reporting guidelines and checklist.

| **Section** | **Pfeiffer** | **Husing** | **Hart** | **Fortner** | **Shi** | **Kitson** | **Choi** | **Bafligil** | **Fritsche** |
| --- | --- | --- | --- | --- | --- | --- | --- | --- | --- |
| **Title** |  |  |  |  |  |  |  |  |  |
| **Abstract** |  |  |  |  |  |  |  |  |  |
| **Introduction** |  |  |  |  |  |  |  |  |  |
| **Methods** |  |  |  |  |  |  |  |  |  |
| **Open Science** |  |  |  |  |  |  |  |  |  |
| **Patient & Public Engagement** |  |  |  |  |  |  |  |  |  |
| **Results** |  |  |  |  |  |  |  |  |  |
| **Discussion** |  |  |  |  |  |  |  |  |  |
| **Overall Quality** |  |  |  |  |  |  |  |  |  |

*Using the TRIPOD guidelines and designated checklist sections^1^, a quality assessment was conducted for each risk model by two independent reviewers. The results are summarized in Additional File 5, Table S5, with green indicating high quality, yellow indicating medium quality, and red indicating low quality.*

Reference:

1. Collins G S, Moons K G M, Dhiman P, Riley R D, Beam A L, Van Calster B et al. TRIPOD+AI statement: updated guidance for reporting clinical prediction models that use regression or machine learning methods *BMJ* 2024; 385 :e078378 doi:10.1136/bmj-2023-078378
